# Supplementary material for: HMGA1 Modulates Gene Transcription Sustaining a Tumor Signalling Pathway Acting on the Epigenetic Status of Triple-Negative Breast Cancer Cells
Source: Cancers (Basel). 2019 Aug 2;11(8):1105. doi: 10.3390/cancers11081105 (PMC6721465; doi:10.3390/cancers11081105)

Supplementary Materials

PRIMARY ANTIBODY (WB and IF)

α-HMGA1—WB: 1:500 (in–house produced rabbit polyclonal antibody)
α-histone H3—WB: 1:4000 (Abcam, #ab1791)
α-histone H3K9me3—WB: 1:1000 (Abcam, #ab8898)
α-histone H3S10ph—WB: 1:1000; IF: 1:100 (Abcam, #ab5176)
α-histone H3S10ph—WB: 1:1000; IF: 1:100 (Abcam, #ab14955)
α-histone H3K14ac—WB: 1:2000 (Abcam, #ab52946)
α-histone H3K27me3—WB: 1:1000 (Cell Signaling Technology, #C36B11)
α-histone H3S28ph—WB: 1:500 (Millipore, #07-145)
α-MSK1—WB: 1:1000 (Cell Signaling Technology, #C27B2)
α-ERK1/2—WB: 1:1000 (Sigma Aldrich, #M5670)
α-phosphoERK1/2—WB: 1:1000 (Sigma Aldrich, #M8159)
α-AURKB—WB: 1:1000 (Novus Biologicals, # NB100-294)
α-RSK2—WB: 1:500; IF: 1:25 (Millipore, #06-918)
α-histone H2B—WB: 1:2000 (Abcam, #ab52985)
α-histone H2BK5ac—WB: 1:500-1:1000; IF: 1:100 (Abcam, #ab40886)
α-histone H2BK16ac—WB: 1:10000 (Abcam, #ab177427)
α-histone H2BK20ac—WB: 1:2000 (Abcam, #ab177430)

SECONDARY ANTIBODY (WB)

α-Rabbit IgG (Whole molecule) Peroxidase conjugate—WB: 1:5000 (Sigma-Aldrich, #A0545)
α-IgG mouse (Whole molecule) Peroxidase conjugate—WB: 1:5000 (Sigma–Aldrich, #A9044)

SECONDARY ANTIBODY (IF)

α-IgG rabbit Alexa Fluor 488—IF: 1/750 (Invitrogen, #A11008)
α-IgG mouse Alexa Fluor 488—IF: 1/750 (Invitrogen, #A11001)
α-IgG rabbit Alexa Fluor 594—IF: 1/750 (Invitrogen, #A11012)
α-IgG mouse Alexa Fluor 594—IF: 1/750 (Invitrogen, #A11005)

siRNA sequences (5' → 3')

CTRL: ACAGTCGCGTTTGCGACTG
HMGA1 (A1_1): GACAAGGCUAACAUCCCACTT
HMGA1 (A1_3): ACTGGAGAAGGAGGAAGAG
RSK2: GCAUUCCACCUAGUGCUAATT
CBP: CCTTCTAGCACCGGTGTAA
EP300: GCAAACAATCGAGCGGAAT
Silencer® Select Negative Control (Life Technologies, #4390846)
MSK1: Silencer® Select Pre-Designed and Validated siRNA (Life Technologies, #4427038-s1769)
MSK2 : Silencer® Select Pre-Designed and Validated siRNA (Life Technologies, #4427038-s17138)

qPCR Primers

GENE name, FORWARD PRIMER (5′-3′), REVERSE PRIMER (5′-3′);
GAPDH, TCTCTGCTCCTCCTGTTC, GCCCAATACGACCAAATCC;
CYC33, CTTCATGCTGCGTTCATTCC, CCTCGGTGCTTTTCTGTTTC;
HMGA1, ACCAGCGCCAAATGTTCATCCTCA, AGCCCCTCTTCCCCACAAAGAGT;
AURKB, TGAGGAGGAAGACAATGTGTGGCA, AGGTCTCGTTGTGTGTGATGCACTCT;
CENPF, TCAGGCAAGAGGCAAAGATCCAGT, TGGCTCAAACTCAGTACCTTCCGT;
KIF23, CCTGAGGGCTACAGACTCAACCGA, TCTGGGTGGTGTGAGTGCCAA;
KIF4A, AAGCCAAACGCCATCTGAATGACC, TTGACCACGCACTTCAGTAAGGGA;

CBP, GGTTTTGTGTGCGACAACTG, TTCCAAGTGGTTTCCCAGTC;
EP300, CAAACGCCGAGTCTTCTTTC, TTGAGCTGCTGTTGGCATAG;
RSK2, GAGAGCGGAAAATGGTCTTC, CAAGCAGCATCATAGCCTTG;
MSK1, TGGAACACATTAGGCAGTCG, ACCTCATGCTCTGTGAAACG;
MSK2, CAAGCTCGGCATCATTACC, TCTTTCTCCTCCGTCAGGAAC;
JUN, ATCAAGTGGCATGTGCTGTG, CCACCAATTCCTGCTTTGAG;
FOS, GTTGTGAAGACCATGACAGGAG, TCCTTTCCCTTCGGATTCTC;
MYC, TTCGGGTAGTGGAAAACCAG, TCGTCGCAGTAGAAATACGG;
ATF3, GGTTTGCCATCCAGAACAAG, CGTCGCCTCTTTTTCCTTTC;
COX-2, TGAGTGTGGGATTTGACCAG, CTGTGTTTGGAGTGGGTTTC;
VIM, CCAGCTAACCAACGACAAAGCCCG, TGCGTTCAAGGTCAAGACGTGCCA
SNAI2, TTCGGACCCACACATTACCTTG, AGGGCAAGAAAAAGGCTTCTCC
LEF1, CGAATGTCGTTGCTGAGTGT, GCTGTCTTTCTTTCCGTGCT

qPCR Primers for ChIP

AURKB -0.8 kb, CAAACAACACGGCAGCTAAC, TCTGACATTCCTGGGAGGAG;
AURKB TSS, TTGGGTTCCCATGACTTACG, CCCGCAAACAACTGAATCTG;
AURKB intragenic, CCAAACTGCTCAGGCATAAC, ATCAGGCGACAGATTGAAGG;
CCNE2 –2.6 kb TSS, (Reference 54)
SERPINE1 promoter, CTGGTATAAAAGGAGGCAGTGG, CGTGTGGGTCTTCTTGACAG
SERPINE1 3'UTR, TTCCCCAATTACAGGGTGAC, TGTCCACTGCTCACACACAG
SERPINE1 Intergenic region 7kb from 3' SERPINE1, CCAGGATTTTTCTCCCTTCC, CTGACTCCAGGATGAAATGC

INHIBITORS

BI-D1870 (Axon MedChem, #1528)
SB747651A (Axon MedChem, #1897)
BIRB796 (Axon MedChem, #1358)
UO126 (Merck Chemicals, #662005)
C646 (SIGMA #SML0002)

Concentrations and incubations time are always reported in the figure legends.

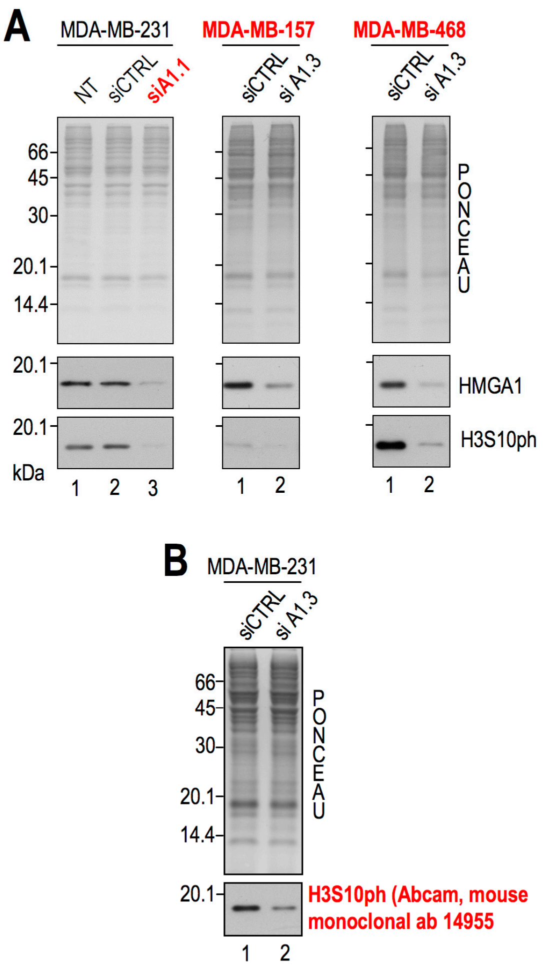

Figure S1. HMGA1 affects H3S10ph: data validation. WB analyses of cells transfected with control (siCTRL) and HMGA1–targeting (siA1_1 or siA1_3) siRNAs. The cell lines analysed are indicated above each WB. The antibodies used are indicated on the right side. Representative red ponceau stained membranes for loading and quantification control are shown. MW markers (kDa) are indicated on the left. In red are evidenced the differences with respect to the data shown in Figure 2.

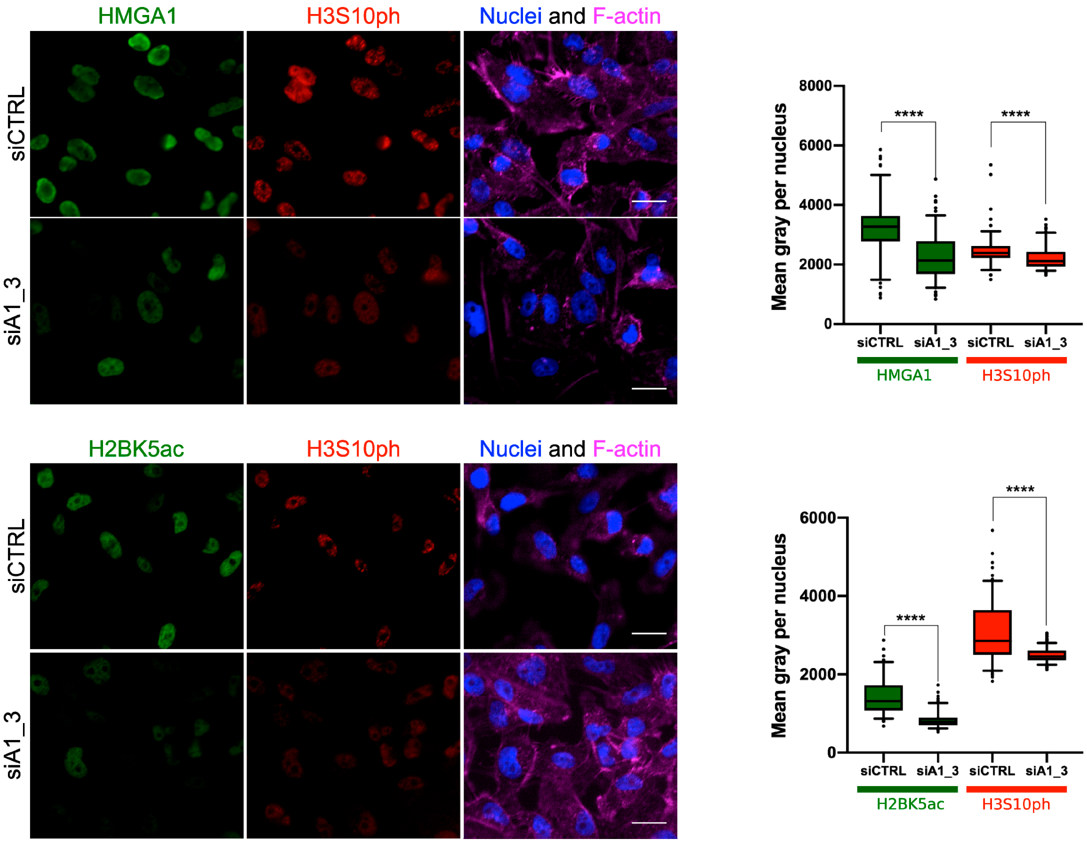

Figure S2. H3S10ph and H2BK5ac are interphasic PTMs down regulated upon HMGA1 silencing**.** Immunofluorescence assays of MDA–MB–231 cells treated with control (siCTRL) or HMGA1–targeting (siA1_3) siRNAs. Representative images are shown. Cells were subjected to immunological detection using α–HMGA1 (rabbit) and α–H3S10ph (mouse) or α–H3S10ph (mouse) and α–H2BK5ac (rabbit) antibodies. Nuclei were stained with Hoechst. Box plots show medians and quantiles distribution of nuclear mean grey values for HMGA1, H3S10ph, and H2BK5ac signals. Whiskers extend to 5-95 percentiles and outliers are represented as dots. Data were analysed with Shapiro-Wilk normality test and Mann Whitney test (**** :$p < 0.0001$).

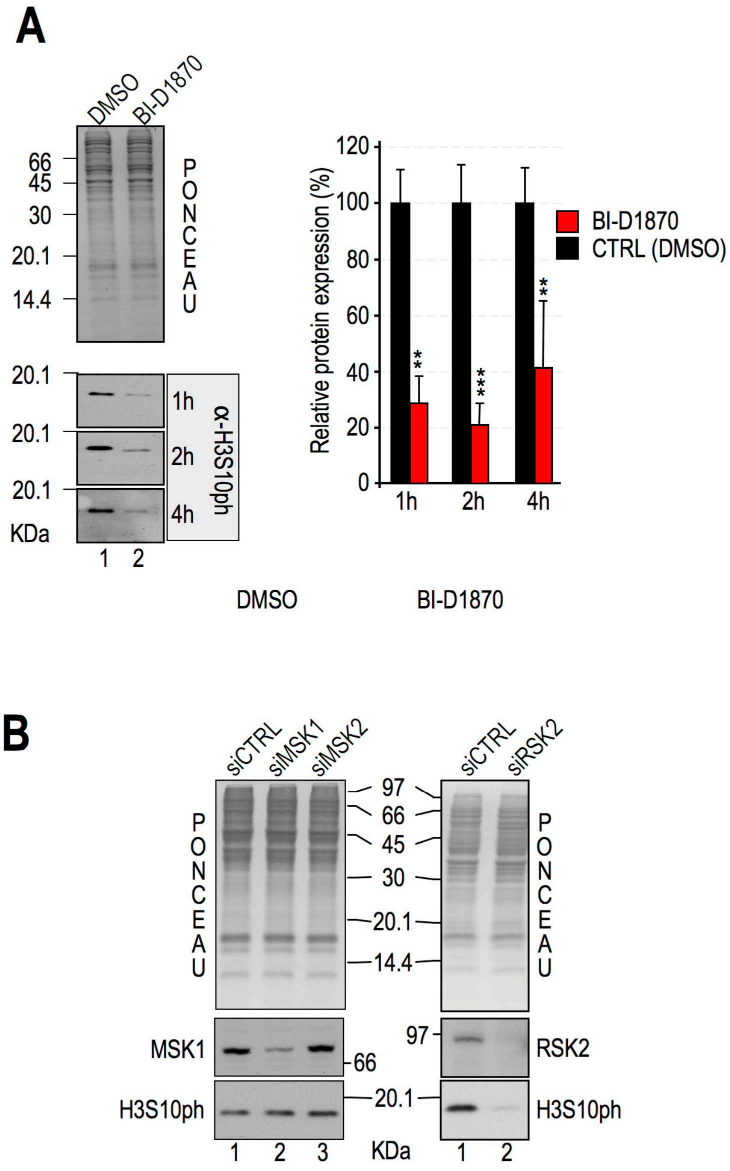

Figure S3. RSK2 (but not MSK1/2) specifically affects the level of H3S10ph**.** (**A**) WB analysis of MDA-MB-231 cells treated with BI–D1870 (10 μM) or with DMSO as a control and harvested after 1, 2, or 4 hours. Cell extracts were analysed for H3S10ph. Densitometric analyses of WB signals are shown on the right part of the panel: means and standard deviations are reported ($n = 3$). ** : $p < 0.01$; *** : $p < 0.001$. (**B**) WB analysis of MDA-MB-231 cells transfected with control (lanes 1), MSK1 (lanes 2) and MSK2 (lanes 3) siRNAs. Cell extracts were analysed for H3S10ph (left side). The same experiment was done silencing RSK2 (right side). α–MSK1 and α–RSK2 antibodies were used to verify the silencing efficacy. Red ponceau stained membranes are shown as loading control. MW markers (kDa) are indicated. All the WB experiments have been performed in biological triplicates providing consistent results. Representative images are shown. .

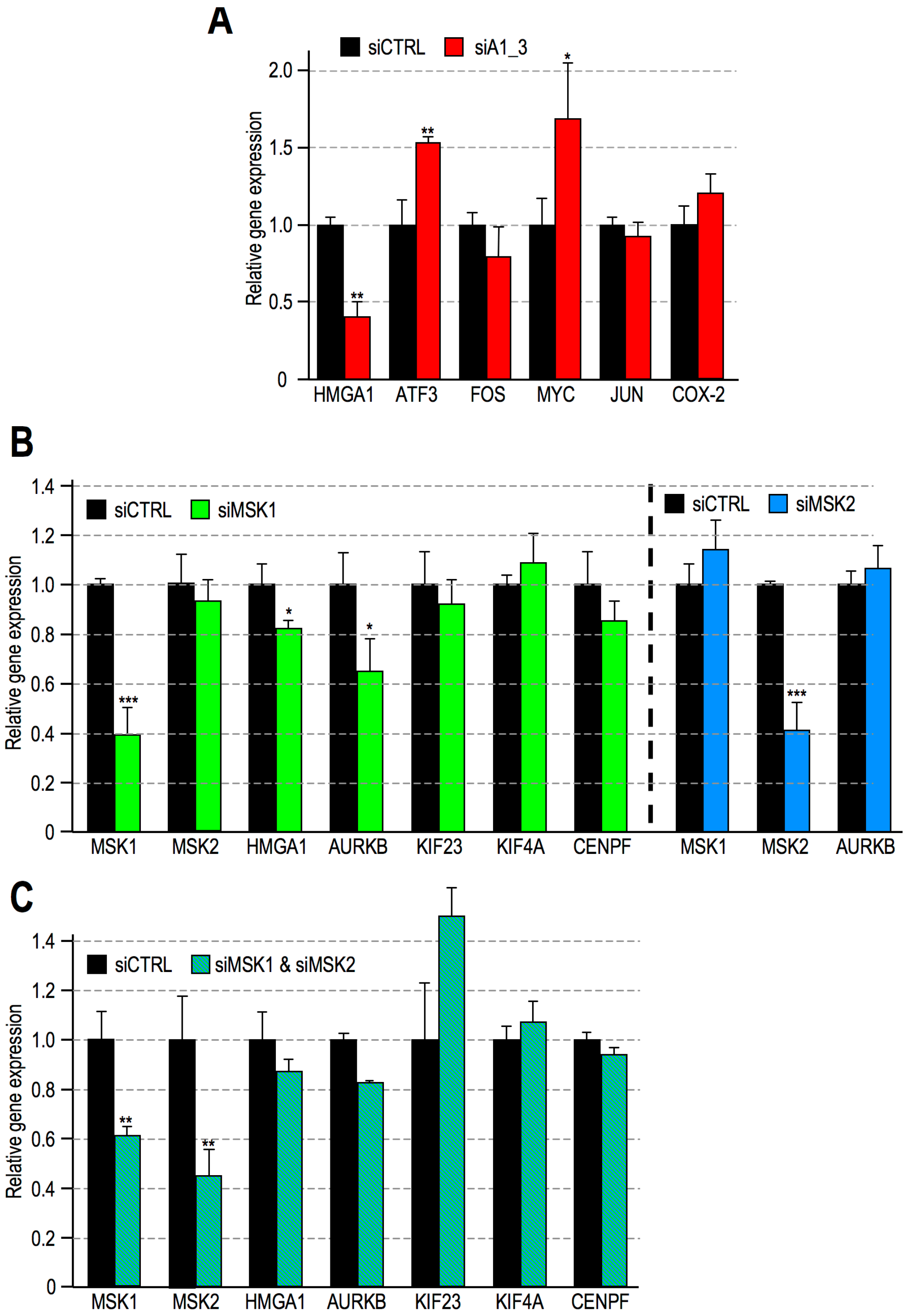

Figure S4. The silencing of RSK2 but none that of MSK1/2 is responsible for the transcriptional regulation of HMGA1–dependent genes. (A) RT-qPCR analysis of MDA-MB-231 cells transfected with control (siCTRL) and HMGA1 (siA1_3) siRNAs and harvested after 72 h. mRNA expression of HMGA1, ATF3, FOS, MYC, JUN, and COX-2 genes was analysed using GAPDH as internal control. (**B**) RT-qPCR analysis of MDA-MB-231 cells transfected with control, MSK1, or MSK2 siRNAs and harvested after 72 h. mRNA expression of MSK1, MSK2, HMGA1, AURKB, KIF23, KIF4A, CENPF was analysed using CYC33 as internal control. Data are the mean relative gene expression values with respect to control samples. Standard deviations (n = 3) and statistical significance (t test) are indicated (p-values: * : $p < 0.05$; ** : $p < 0.01$; *** : $p < 0.001$). (C) Same data with respect to those shown in panel C but MSK1 and MSK2 were contemporary silenced.

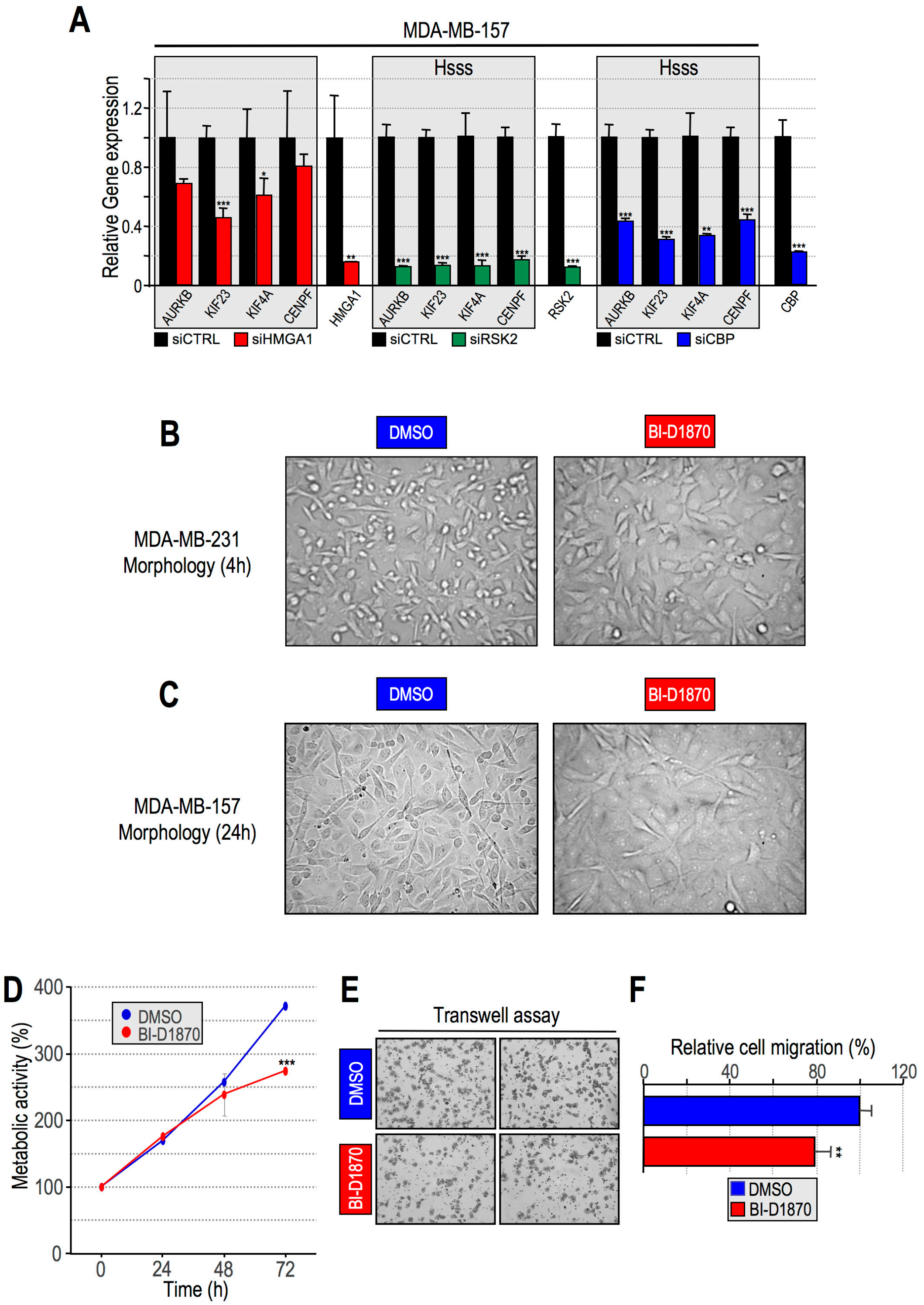

Figure S5. RSK2 and CBP regulate the Hsss in MDA-MB-157 cells. The RSKs inhibitor BI-D1870 modulates MDA-MB-157 aggressiveness features. (**A**) RT-qPCR analysis of MDA-MB-157 cells transfected with control (siCTRL), HMGA1 (siA1_3), RSK2 (siRSK2), and CBP (siCBP) siRNAs and harvested after 72 h. mRNA expression of HMGA1, RSK2, CBP, and of genes of the Hsss (AURKB, KIF23, KIF4A, and CENPF) was analysed using GAPDH as internal control. Standard deviations and statistical significance (*t*-test) are indicated (n = 3). (**B**) Optical microscope images of MDA-MB-231 cells treated for 4 h with BI-D1870 10 μM. DMSO–treated cells are shown as a control (CTRL). (**C**) Optical microscope images of MDA-MB-157 cells treated for 24 h with BI-D1870 10 μM. DMSO–treated cells are shown as a control. (**D**) MTS assay of MDA-MB-157 cells treated with BI-D1870 10 μM in comparison with DMSO treatment as a control. (**E**) Transwell

assay of MDA-MB-157 cells treated with BI-D1870 10 μM. DMSO–treated cells are shown as a control. Representative images are reported. (F) Quantitative evaluation of the Transwell assay (n=4, technical quadruplicate). Standard deviations and statistical significance (t-test) are indicated (p-values: * : $p < 0.05$; ** : $p < 0.01$; *** : $p < 0.001$).

WB Raw data - in red are underlined the lanes shown in the manuscript

FIG1 - left side

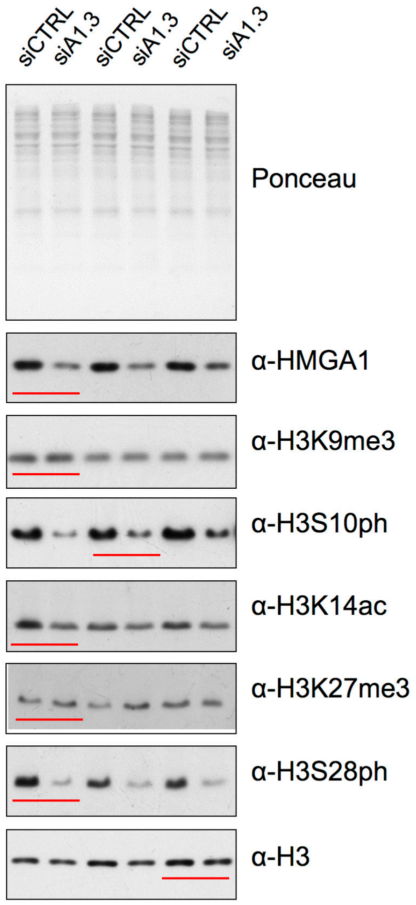

WB Raw data - in red are underlined the lanes shown in the manuscript

FIG1 - right side

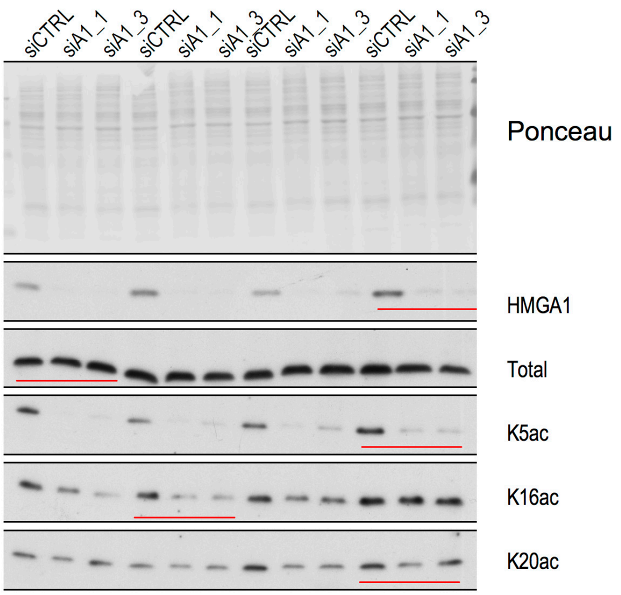

WB Raw data - in red are underlined the lanes shown in the manuscript

FIG2 - panel A

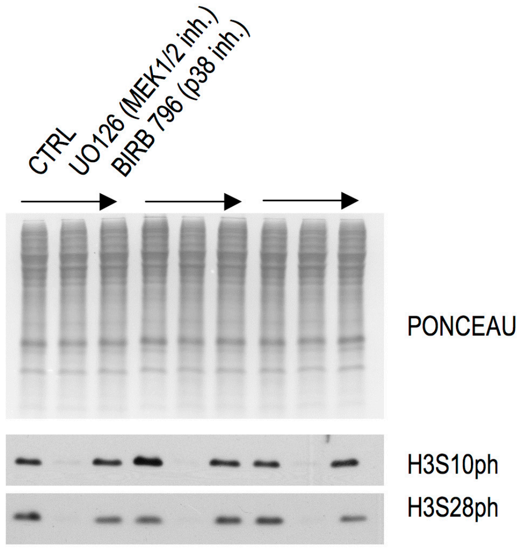

FIG2 - panel B

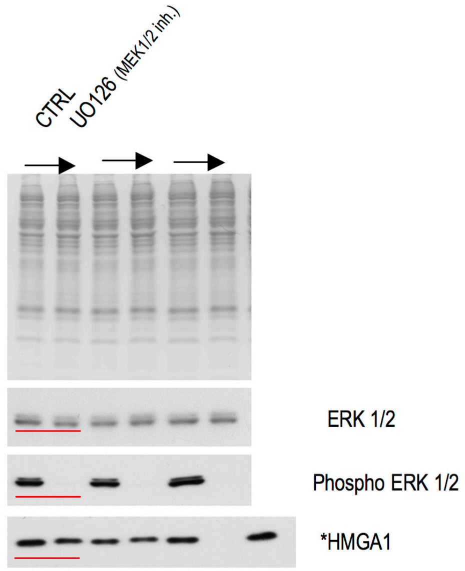

*There was a loading error: we skipped a lane in the gel used for HMGA1 western blot. For this reason there is an empty lane.

WB Raw data - in red are underlined the lanes shown in the manuscript

FIG2 - panel C

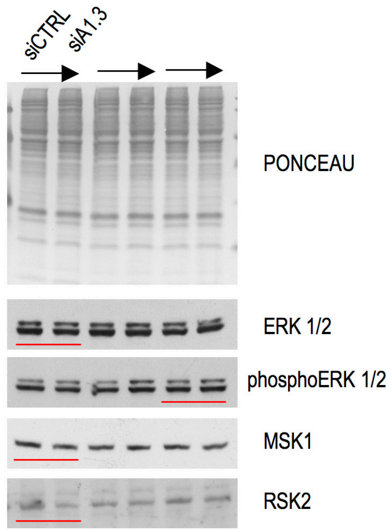

WB Raw data - in red are underlined the lanes shown in the manuscript

FIG2 - panel D

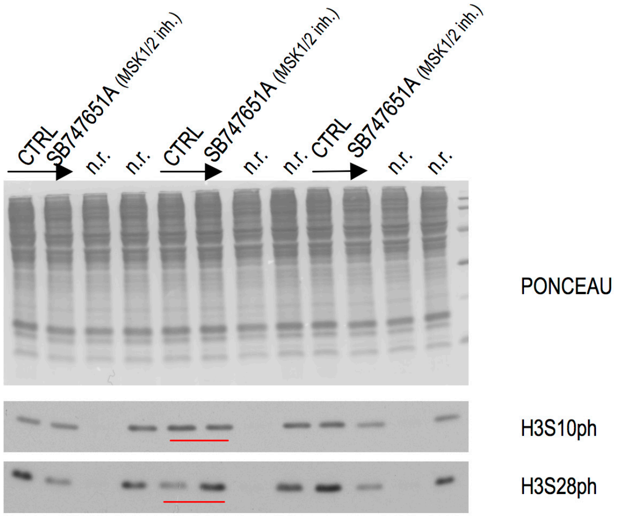

In this experiment we tested also different inhibitors not discussed within this manuscript. The lanes in which those sample have been loaded are named as n.r. (not. related)

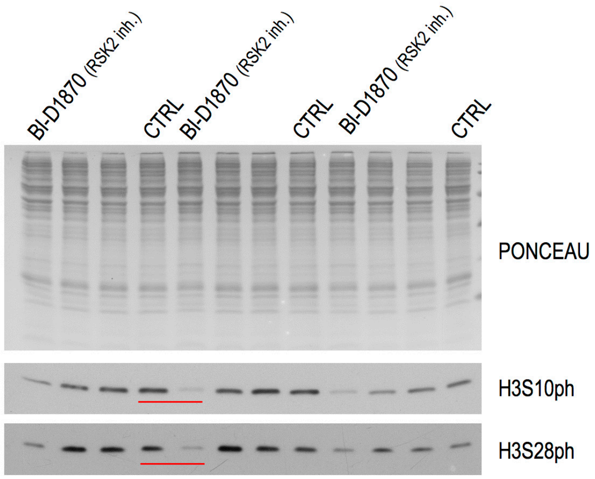

In this experiment we tested different concentrations of BI-D1870 (10, 1, and 0.1 microM) and therefore the loading order is 10 - 1 - 0.1 - CTRL (from left to right - triplicate). The image was done considering only the CTRL and the BI-1870 10 microM concentration.

WB Raw data - in red are underlined the lanes shown in the manuscript

FIG5 - panel A,B, and C

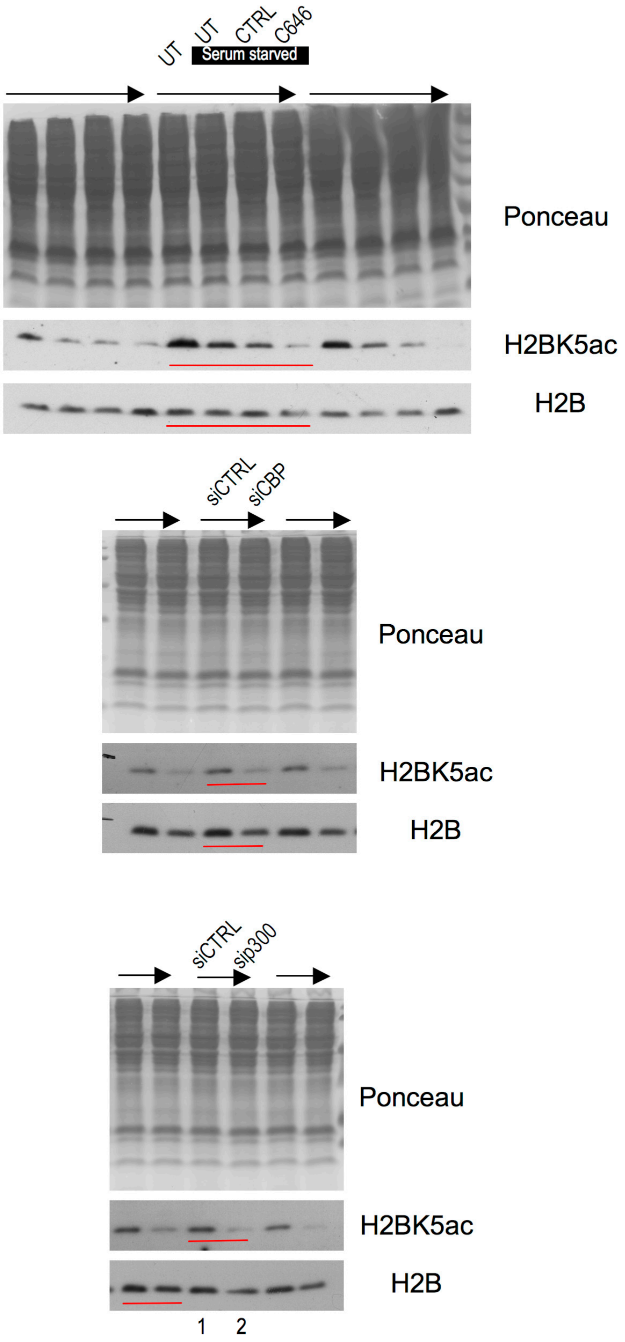

WB Raw data - in red are underlined the lanes shown in the manuscript

FIG6 - panel A

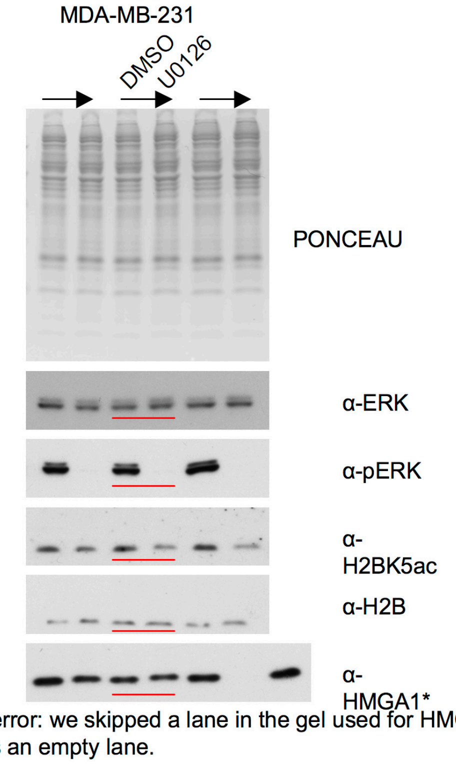

*There was a loading error: we skipped a lane in the gel used for HMGA1 western blot. For this reason there is an empty lane.

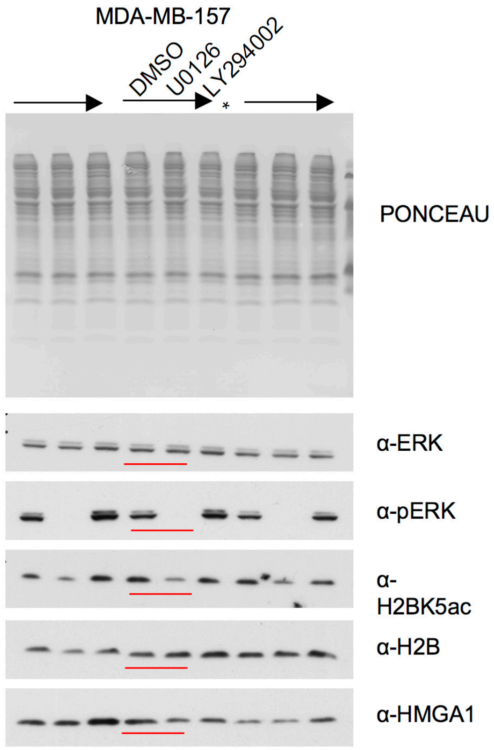

*The inhibitor LY294002 was not included in the results presented in this article

WB Raw data - in red are underlined the lanes shown in the manuscript

FIG6 - panel B

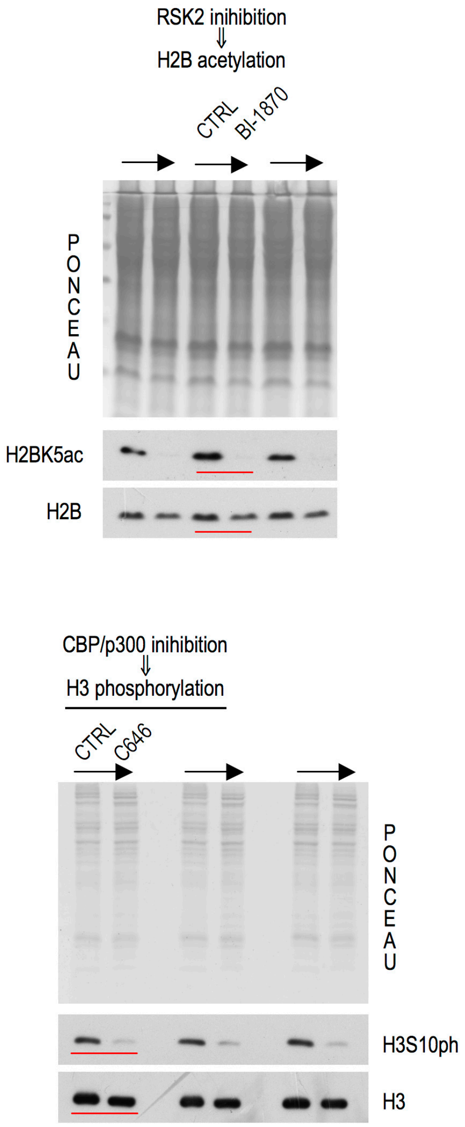

WB Raw data - in red are underlined the lanes shown in the manuscript

FIG7 - panel B

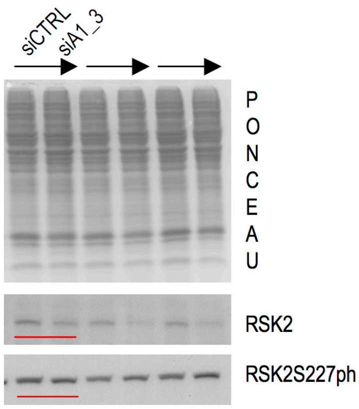

Supplement: Supplementary file 1 [file cancers-11-01105-s001.pdf]
